# Anaotmical variability in the position of cystic artery during laparoscopic visualization

**DOI:** 10.1186/s12893-021-01270-8

**Published:** 2021-05-27

**Authors:** Omer Fateh, Muhammad Samir Irfan Wasi, Syed Abdullah Bukhari

**Affiliations:** 1 Department of Surgery, Sindh Government Qatar Hospital, Karachi, Pakistan; 2 Department of Neurosurgery, Hull University Teaching Hospitals NHS Trust, Hull, UK

**Keywords:** Laparoscopy, Cholecystectomy, Cystic artery variation, Cystic duct, Iatrogenic injury

## Abstract

**Background:**

The laparoscopic view of extrahepatic biliary tract and cystic artery is different anatomically from open approach. Consequently iatrogenic injuries due to inadverent damage to cystic artery are not uncommon. These complications can be prevented by careful dissection in Calots triangle and better knowledge of laparoscopic anatomy of cystic artery and its variations. The aim of this study is to establish the prevalence of variation in position of cystic artery in relation to cystic duct. This will help identify the safe area for dissecting peritoneum in Calots triangle and thus help young surgeons overcome the long learning curve associated with laparoscopy.

**Materials and methods:**

During a 10 year period from January 2009 to January 2019, 1850 laparoscopic cholecystectomies that were performed at a tertiary care hospital were studied. Patients with history of previous abdominal surgery were excluded from the study. Cystic artery was divided into four groups based on its relative position to cystic duct. It includes superomedial, superolateral, anterior and absent cystic artery relative to the cystic duct.

**Results:**

Out of 1850 cases of laparoscopic cholecystectomy 1676 (90.59%) patients had cystic artery superomedial to cystic duct and 96 (5.19%) had a cystic artery at superolateral position to cystic duct. In 48 (2.59%) patients it was found anterior to cystic duct and in 30 (1.62%) patients it was absent.

**Conclusions:**

It is concluded that the most common position of cystic artery is superomedial while the least common position was found to be anterior to cystic duct. Hence it is postulated that blind dissection from anterior side is the safest approach to avoid injury to cystic artery.

## Introduction

Cholelithiasis is one of the most common and prevalent surgical pathology all over the world [1–3]. Traditionally open cholecystectomy has been the treatment of choice for many years but the introduction of laparoscopic technique has revolutionised the management of cholelithiasis. Although it has a long learning curve, yet it is now the standard procedure for symptomatic cholelithiasis as it offers significant benefits over open technique [4, 5].

Despite its advantages laparoscopic cholecystectomy was initially associated with a higher number of iatrogenic bile duct injuries and arterial haemorrhages [6–8]. This was due to the fact that a surgeon who is more familiarized with open technique has to deal with novel anatomical relations. The learning curve of the procedure poses additional difficulties for the surgeon.

In addition to adequate anatomical knowledge of associated regions, proper knowledge and identification of Calots triangle is essential for laparoscopic cholecystectomy.

J.F. Calot in 1891 described a triangular area comprised of the cystic duct, right hepatic duct, and lower edge of the liver [9]. Rocko et al. in 1981 described the possible variations in the region of Calot's triangle [10]. In 1992, Hugh et al. suggested Calot's triangle should be renamed as hepatobiliary triangle, with the small cystic artery branches supplying the cystic duct being called Calot's arteries [11]. The cystic artery typically arises from the right hepatic artery in 70–80% of cases and courses within the cystohepatic triangle to the right of the common hepatic duct [11–13]. Bleeding from cystic artery is a very troublesome complication during laparoscopic cholecystectomy as it decreases the overall visibility in abdomen. Agrusa et al. have reported the incidence of conversion to open surgery because of blood vessel injuries to be 1.2% [14]. V. Kudurupaka reports this to be as high as 6.62% [15].

Young surgeons who have recently started using laparoscopic approach often struggle with approaching the Calot’s triangle. Iatrogenic injuries in this region contribute to a major percentage of morbiditiy in patients treated with either open or laparoscopic cholecystectomy [16]. So it is the prime focus for research in laparoscopic cholecystectomy for the classification of various structures in Calots triangle.

During laparoscopic cholecystectomy gall bladder is mobilized to achieve critical view of safety. This maneuver pulls the structures related to gall bladder and changes the anatomy. The aim of this study is to find the prevalence of positional variations in cystic artery as seen in laparoscopic view in a local population.

This study can help identify safe area for dissecting peritoneum in Calots triangle. It can also play its part in decreasing the incidence of vascular complications in laparoscopic cholecystectomy and help young surgeons with minimal experience overcome the long learning curve associated with it.

## Materials and methods

This is a prospective, sequential, non-randomized, descriptive study which was conducted at a tertiary care hospital within duration of ten years from 2009 to 2019. All patients who were admitted with the diagnosis of cholelithiasis or acute cholecystitis and underwent laparoscopic cholecystectomy were included in this study during the specified duration. All patients with a history of previous upper abdominal surgeries were excluded from this study.

After approval from ethical committee, informed consent was taken from patients. All patients underwent routine investigations and ultrasound of whole abdomen prior to surgery. Complete blood count, liver function test were done pre-operatively. Three surgeons performed the surgeries in a randomised fashion. All surgeries were carried out under general anaesthesia. Pneumoperitoneum was created by Veress needle. A standard 4 port approach was used to proceed with cholecystectomy with two 10 mm trocars (umblicus and mid epigastrium) and two 5 mm trocars (along the right costal margin). Olympus laparoscope with 0 degree camera was used. Visualisation of Calots triangle and variations in position of cystic arteries were visualised on medical grade monitor and duly noted in the prescribed performa. Variations in the position of cystic artery were classified into 4 groups as shown in Table 1.

**Table 1:** Anatomical groups of cystic artery variation in relation to cystic duct and their prevalence

Group	Position of cystic artery in relation to cystic duct	Prevalence (%)
1	Superomedial	90.59
2	Posterolateral	5.19
3	Anterior	2.59
4	Absent	1.62

Sample size was calculated using WHO sample size calculator with one-sided hypothesis test mode formula. The level of significance was 5% while the power of the test was set to be 95%. Non probability consecutive sampling technique was used thus nullifying the selection bias. As per the reference study [17], the test value of population incidence rate for a superomedial cystic artery was found to be 88%. To test the hypothesis of incidence rate in the local population, anticipated population incidence rate was 95%. The authors were interested in rejecting the null hypothesis only if the incidence rate was higher than 88%. The sample size was calculated to be 1850.

All the variations were recorded using performa and were statistically analysed via SPSS version 23.

Patients were discharged on the 1st postoperative day on analgesics if uncomplicated.

## Results

A total of 1850 patients were included in the study that underwent laparoscopic cholecystectomy during the specified duration. All patients included in the study underwent routine preoperative investigations including LFTs (Liver Function Tests) and ultrasound upper abdomen.

In this study 1658 patients (89.62%) were females and 192 (10.37%) were male. Minimum age was found to be 19 years and maximum age was 46 years with the mean being 30.39. The standard deviation was found to be 6.098.

Out of these patients 38 (2.05%) were diagnosed as acute cholecystitis and 1782 (96.32%) were diagnosed as cholelithiasis. Mucocele of bladder was diagnosed in 17 (0.91%) patients and 13 (0.7%) patients were diagnosed as empyema gallbladder.

Among the 1850 patients operated for laparoscopic cholecystectomy, 27 (1.46%) were converted to open cholecystectomy while the rest were carried out successfully without complication.

**Fig. 1:**
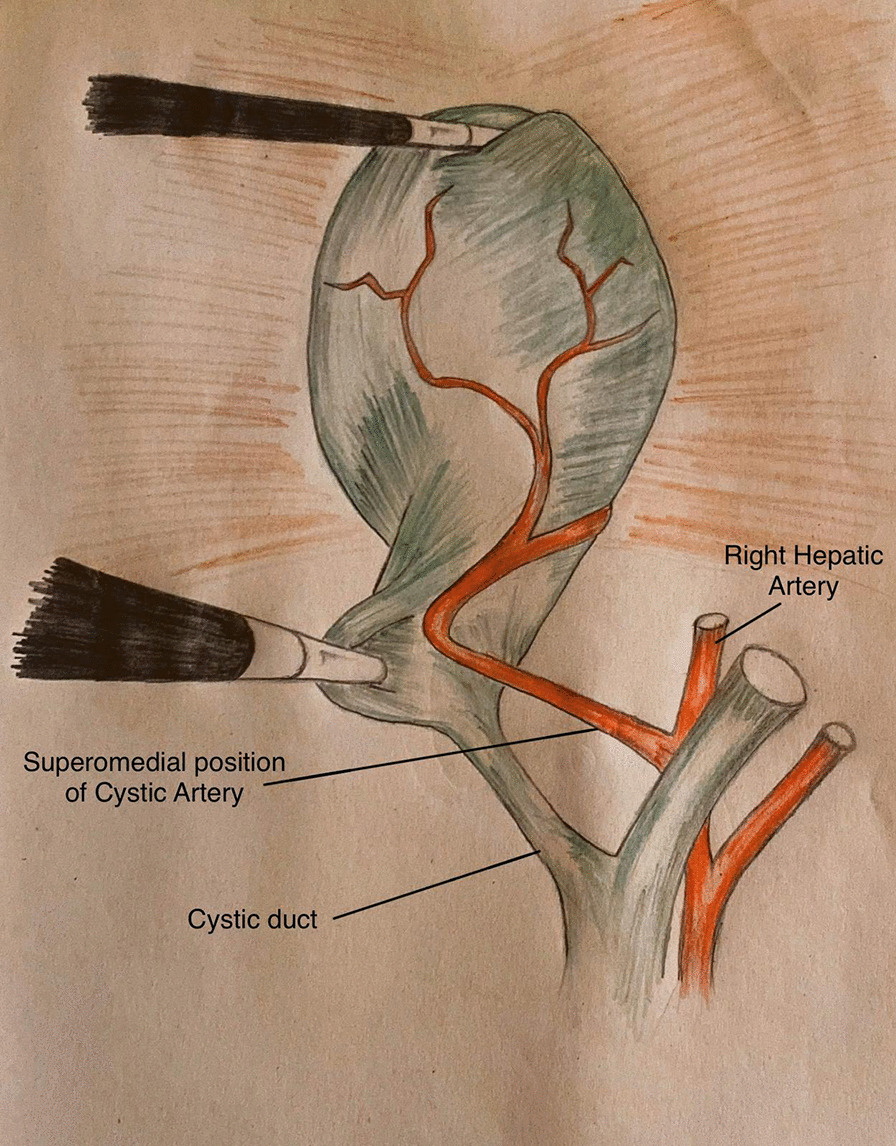
Position of cystic artery in relation to cystic duct; Superomedial

**Fig. 2:**
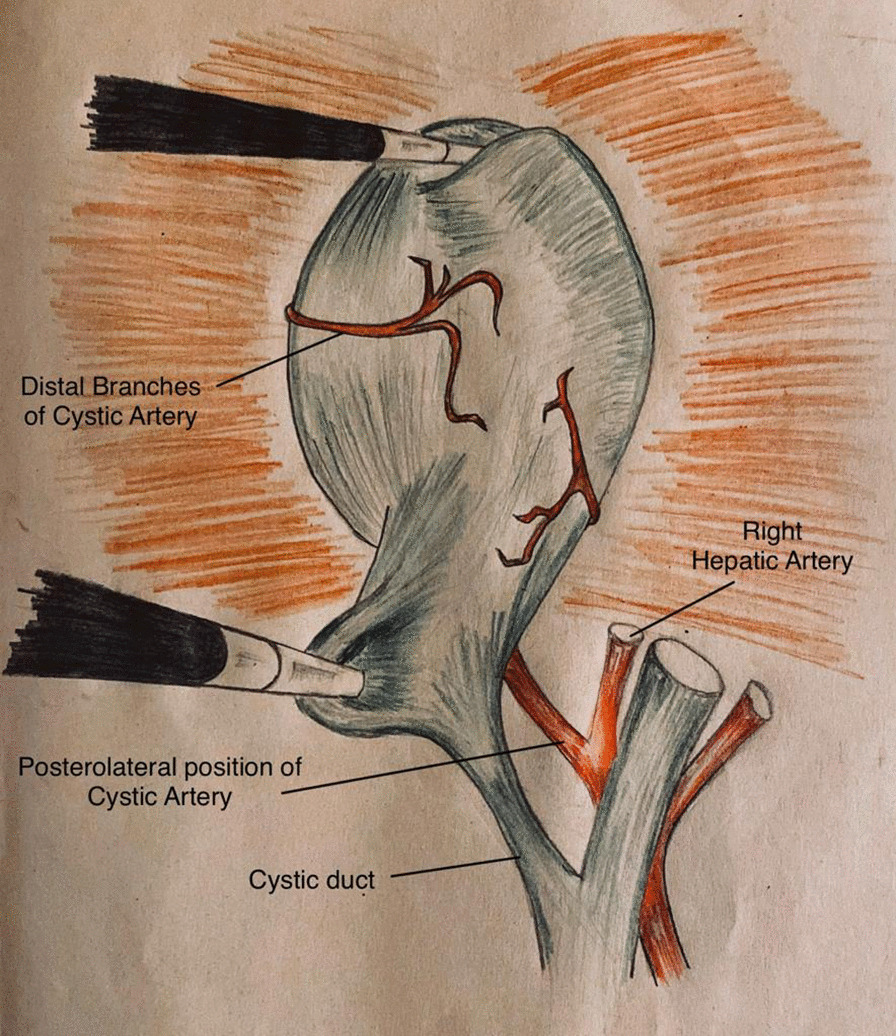
Position of cystic artery in relation to cystic duct; posterolateral

**Fig. 3:**
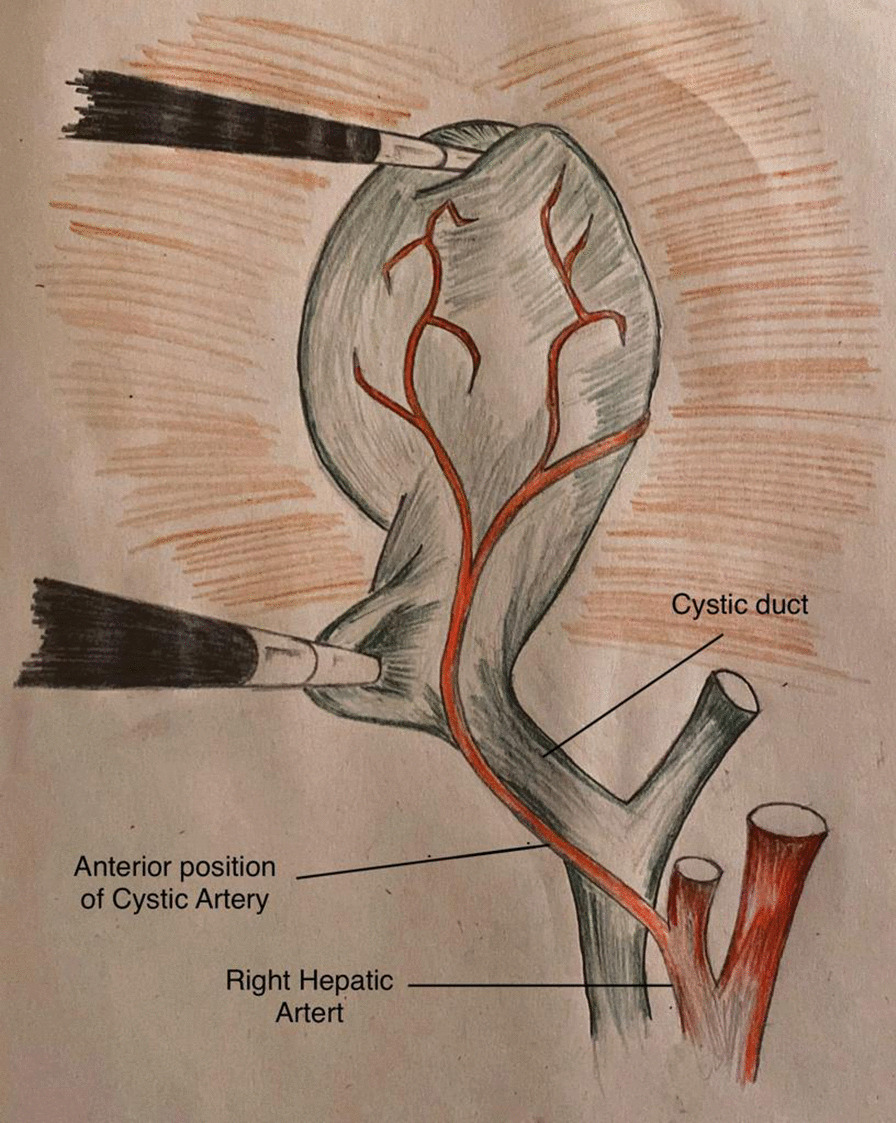
Position of cystic artery in relation to cystic duct; Anterior

The study revealed that 1752 patients (94.7%) had cystic artery at superomedial position to cystic duct and were included in group 1 (Fig. 1). The second most common position was found to be cystic artery posterolateral to cystic duct in 10 (5.208%) patients and included in group 2 (Fig. 2). In 5 (2.6%) patients cystic artery was found to be at anterior position to cystic duct (Fig. 3) and absent in 3 (1.56%) patients thus included in group 3 and 4 respectively.

## Discussion

Laparoscopic cholecystectomy is the gold standard technique for treatment of symptomatic cholelithiasis and acute cholecystitis. However, it has a high risk of iatrogenic injury to bile duct and cystic artery [8]. To avoid such iatrogenic injury to bile duct and vascular structures, a surgical strategy was advised by Strasberg et al. [18] involving three steps. First step is blind dissection of Calots triangle including hepatoduodenal ligament. Second step involves mobilisation of lower part of gallbladder. Third step include identification and isolation of two main structures that is cystic duct and artery. This forms the basis for the infundibular approach for removing gall bladder with cauterisation from neck upwards. This is the most common approach used for removing gall bladder and provides a good visual access to the surgeon [19]. This strategy is known among surgeons as the critical view of safety.

The first step in critical view of safety is the most significant part of the procedure as it deals with the blind dissection in Calots triangle. As with any other blind procedure, this blind dissection poses risk of vascular damage as the relevant artery is not visible during this step. This vascular damage causes obstruction of field of vision leading to increased risk of further iatrogenic injury to biliary tree. All this menace may result in conversion to open cholecystectomy.

The course, length and position of cystic artery are highly variable and are thus prone to iatrogenic injury. In order to avoid such complication it is essential to perform careful blunt dissection in Calots triangle during laparoscopic and open cholecystectomy. In this study we aim to study the variations in cystic artery anatomy with relation to the cystic duct. This can help to establish an area of dissection least likely to encounter cystic artery. We found that superomedial was the most common position (90.62%) in relation to cystic duct and the least common position was found to be anterior (2.6%). It was found absent in 3 (1.56%) patients. In view of these results we can postulate that it is safe to dissect the peritoneum anterior to the cystic duct as this is the area with least prevalence of cystic artery.

Variation in anatomy of cystic artery has been studied in several studies previously. A number of prominent and historic studies on this topic are from an age when CT and MR imaging was not performed routinely and laparoscopy was not common [10, 20, 21]. In a study conducted by M. Taimur et al. [16] in 2011 the most common position of cystic artery was found to be superomedial (88%) and least common position was posterior (3%) while anterior was found to be in 6% of patients. M Ayyaz et al. [22] have reported the anterior position of cystic artery in 15% of patients. These studies show a much higher frequency in the anterior region than our study. However our study was conducted on a larger sized population and comparable sample size.

A systematic meta-analysis by R.G Andall et al. [23] reviewed 9800 cases and presented their results for variation of cystic artery. This review was not specific for laparoscopic patients and the positional variation was not defined as per laparoscopic view. However, they reported cystic artery to be multiple in 8.9% and absent in 0.34% cases. Cystic artery was found anterior to the Common bile duct in 5.9% cases while inferior in to the cystic duct in 4.9% cases. A significant contrast with our study is with the variation where cystic artery lies anterior to cystic duct which has been reported by Andall et al. to be 33.8% while it is the least common variation in our study (only 2.9%). Our study was conducted specifically on laparoscopic patients and the anatomy was documented after achieving critical view of safety, we postulate that it should be considered the least common variation during a laparoscopic procedure.

Milivoj Balija et al. [24] have described the variations in cystic artery during laparoscopic visualization and is most comparable with our study. They reviewed 1000 cases and found that 4.5% of patients have a cystic artery anterior to the cystic duct. Other studies in literature have reported this variation in the range of 2–30% [10, 25, 26].

Our study was conducted specifically on laparoscopic patients and the anatomy was documented after achieving critical view of safety. After reviewing literature and comparing the results of our study, we postulate that anterior position of cystic artery in relation to cystic duct should be considered the least common anatomical variation during laparoscopic visualization.

The thorough knowledge of anatomy of extrahepatic biliary tract arterial supply and its variation is crucial. This knowledge helps in reducing the unwanted bleeding that might result in obscuring the vision causing damage to other biliary and vascular structures. Iatrogenic injury to common bile duct and cystic artery can be avoided by careful dissection at Calots triangle and hepatoduodenal ligament [27].

It is thus essential to establish a safe zone for the blind dissection where the cystic artery can be present least likely. This study provides evidence for this step to be carried out safely without bleeding the cystic artery. As a first step in achieving critical view of safety, blind dissection in Calots triangle can be safely started anterior to the cystic duct thus reducing the chance of injury to cystic artery. Young surgeons can benefit from these findings in overcoming fear of complications and the learning curve associated with laparoscopic approach.

### Disclosure

A preprint containing 2 years data from 2018–2019 was published online [28].

## Conclusion

It is concluded in this study that during laparoscopic cholecystectomy the most common position of cystic artery is superomedial while the least common is anterior to cystic duct. In view of the results from this study we postulate that blind dissection in Calots triangle, as the first step to achieve critical view of safety, should thus be performed anterior to the cystic duct as it is least likely to encounter cystic artery and cause iatrogenic injury.

## Data Availability

The datasets used and/or analysed during the current study available from the corresponding author on reasonable request.
